# PITX1 as a grading, prognostic and tumor-infiltrating immune cells marker for chondrosarcoma: a public database-based immunoassay and tissue sample analysis

**DOI:** 10.3389/fonc.2025.1477649

**Published:** 2025-04-11

**Authors:** Zikun Huang, Dongchen Liu, Ying Zhang, Weiqing Lu, Lan Hu, Jinghao Zhang, Lei Xie, Shubiao Chen

**Affiliations:** ^1^ Department of Orthopaedics, First Affiliated Hospital of Shantou University Medical College, Shantou, China; ^2^ Department of Pathology, Shantou University Medical College, Shantou, Guangdong, China; ^3^ Department of Radiotherapy, Cancer Hospital of Shantou University Medical College, Shantou, Guangdong, China; ^4^ Clinical Research Center, Cancer Hospital of Shantou University Medical College, Shantou, Guangdong, China; ^5^ Sport Medicine Centre, First Affiliated Hospital of Shantou University Medical College, Shantou, Guangdong, China; ^6^ Department of General Surgery, First Affiliated Hospital of Shantou University Medical College, Shantou, China

**Keywords:** tumor grade, PITX1, chondrosarcoma (CHS), tumor-infiltrating immune cell (TIC), immune targets

## Abstract

**Background:**

Chondrosarcoma (CHS) is a rare bone cancer originating from chondrocytes, with high-grade cases associated with high mortality rates. However, the prognostic factors and therapeutic targets for CHS have not been studied.

**Methods:**

Graded gene differential analysis was conducted on 97 CHS tissues to identify genes associated with CHS grading. Additionally, we performed GO and KEGG enrichment analyses of the differentially-expressed genes (DEGs), as well as GSEA analysis, differential expression analysis, survival analysis, and univariable and multifactorial COX analysis of paired-like homology structural domain transcription factor 1 (PITX1). Furthermore, our findings investigated the relationship between tumor-infiltrating immune cells (TICs) in CHS tumors using CIBERSORT to calculate proportions and differences. Our findings also explored the associations among gene expression patterns, survival prognosis, TICs, and immune checkpoints across various cancer types. Finally, immunohistochemical staining was carried out on self-collected clinical samples to assess PITX1 expression levels and correlate them with clinical information.

**Results:**

Gene differential expression analysis revealed a strong correlation between PITX1 expression and tumor grade. GO, KEGG enrichment, and GSEA analysis demonstrated the association of PITX1 with cell proliferation-related processes, such as cell cycle regulation and mitosis, and differentiation-related processes, such as RNA processing. PITX1 expression was associated with tumor stage and survival outcomes. Immunoassay indicated a positive correlation between PITX1 levels and TICs, immune checkpoints, and graded TICs. Pan-cancer analysis confirmed the differential expression of the PITX1 gene across multiple cancers, impacting survival prognosis, TIC patterns, and immune checkpoint regulation. Lastly, our 75 collection of clinical patient tissue samples exhibited varying levels of PITX1 expression across different cancer grades while also demonstrating a significant association with tumor differentiation and metastasis.

**Conclusion:**

PITX1 is a novel biomarker for distinguishing between high-grade and low-grade CHS, serving as a prognostic indicator for patients with this condition and presenting a promising target for immunotherapy. These findings offer innovative insights into the treatment of CHS.

## 1 Introduction

Chondrosarcoma (CHS) is one of the most common bone sarcomas, and is characterized by malignant cells producing a cartilaginous matrix (1, 2). It accounts for 25% of all bone sarcomas, and typically affects adults 30 to 60 years of age, and has a preference for the proximal femur and pelvis (2–5). The current standard treatment for osteochondroma (OC) is surgical resection unless the skeleton is still immature (6). Malignant transformation of OC leads to low-grade CHS, occurring in approximately 0.5–5% of patients (7, 8). Understanding the mechanism behind the progression from low to high grade, and distinguishing between them, remains an urgent and challenging task (9). Low-grade CHSs are locally aggressive but rarely metastasize (10). However, high-grade CHSs often metastasize and have a poor 10-year survival rate of around 30% (11, 12).

Multiple genetic and epigenetic alterations have been observed in cancer progression (13). Over the past few decades, several biomarkers, including Ki67, SOX4, SOX9, IDH, HIF1a, and AKT have been identified as being upregulated in CHS. Various efforts have been made to discover reliable molecular markers capable of distinguishing high-grade from low-grade CHS, such as Galetion-1 and MMP-1. However, no molecular marker has definitively demonstrated an ability to differentiate between high-grade and low-grade CHS.

Paired-like homeodomain transcription factor 1 (PITX1) was initially identified as a bicoid-related transcription factor involved in expression of the proopiomelanocortin gene (14). PITX1 plays a crucial role in determining hindlimb morphology in vertebrates and is predominantly expressed in the hindlimb (15). Additionally, PITX1 is responsible for the patterning and development of hindlimb cartilage and long bones. Deletion of the mouse PITX1 gene hampers hindlimb cartilage and bone development (16). Recent studies provide further evidence implicating PITX1 as a tumor suppressor that correlates with patient survival and metastasis in various parenchymal tumors, including gastric carcinoma, breast cancer, esophageal carcinoma, lung cancer, and osteosarcoma (17–22). Our previous investigation demonstrated downregulation of PITX1 expression in osteosarcoma tissues was associated with poor survival of osteosarcoma patients. However, the roles of PITX1 in CHS remain poorly understood.

In this study, we conducted bioinformatic analysis on a cohort of 97 CHS tissues to investigate CHS histological grade, survival rate, TIC patterns, and immune checkpoint expression profiles. Furthermore, we utilized our own collected samples of CHS and OC tissues to evaluate PITX1 expression levels and explore correlations between PITX1 expression and clinicopathological variables among these patients.

## 2 Materials and methods

### 2.1 Patient samples

In this study, primary CHS and adjacent non-tumorous tissues were collected from patients who underwent radical resection at the First Affiliated Hospital of Shantou University Medical College from 2002 to 2014. This included 35 cases of CHS and 35 cases of OC. The CHS group consisted of 22 men and 13 women, with a median age of 40 years (range: 13 to 67 years), while the OC group included 18 men and 17 women, with a median age of 17 years (range: 1 to 78 years) (**Table 1**). All patients underwent potentially curative surgery without preoperative chemotherapy or radiotherapy. Additionally, as negative controls, normal lower limb cartilage tissues were collected from 5 femoral head cartilage samples obtained from different patients who had suffered femoral neck fractures due to trauma. All tissues were fixed in formalin and embedded in paraffin. Written informed consent was obtained from all participants involved in the study. This study was approved by the Ethics Review Committee of the First Affiliated Hospital of Shantou University Medical College(B-2024-138).

**Table 1:** Clinical parameters of CHS, OC and normal cartilage patients.

Variables	Chondrosarcoma	Osteochondroma	Normal cartilage
**N**	35	35	5
Age, year, n%			
<30	7 (20)	26 (74.3)	3 (60)
30~59	23 (65.7)	5 (14.3)	1 (20)
≧60	5 (14.3)	4 (11.4)	1 (20)
Gender, n%			
Male	22 (62.9)	18 (51.4)	3 (60)
Female	13 (37.1)	17 (48.6)	2 (40)
Grade, n%			
Low-grade	23 (65.7)	**-**	**-**
High-grade	12 (34.3)	**-**	**-**
Location, n%			
Pelvis	7 (20)	**-**	**-**
Lower limb	12 (34.3)	24 (68.6)	5 (100)
Upper limb	4 (11.4)	9 (25.7)	**-**
Skull	7 (20)	**-**	**-**
Others	5 (14.3)	2 (5.7)	**-**
Metastasis, n%			
Yes	6 (17.1)	**-**	**-**
No	29 (82.9)	**-**	**-**

### 2.2 Immunohistochemistry

All tissues were deparaffinized and hydrated, then heated in 1% sodium citrate buffer (pH = 6.0) by microwave for 30 minutes and cooled to room temperature for 1 hour. The specimens were treated with 0.3% hydrogen peroxide and blocked with 5% BSA for 30 minutes, then incubated overnight with PITX1 antibody (Abcam, Cambridge, MA, USA, 1:200) at 4°C. After washing, sections were incubated with biotinylated anti-rabbit IgG at room temperature for 1.5 hours, then counterstained with 0.1% hematoxylin. The staining intensity was evaluated by IPP software (Image Pro Plus software version 6.0) on 10 randomly selected visual fields from each section. Omission of the primary antibody served as a negative control, and the results were expressed as the mean integral optical density (IOD). Staining intensity not above background level, slightly above background, and significantly above background were considered negative, weak expression, and strong expression, respectively. An average IOD that was 50% higher than the average IOD of normal cartilage tissue was considered strong expression, and an average IOD that was 50% lower than the average IOD of normal cartilage tissue was considered weak expression. Both weak and strong expressions were considered positive.

### 2.3 Dataset download and data preprocessing

Transcriptome RNA-seq data (E-MTAB-7264) of 164 tumor samples from 102 cartilage tumors and the corresponding clinical data were downloaded from EMBL’s European Bioinformatics Institute (EMBL-EBI). Among these, 97 samples with clinical data on “Grade” and “Survival information” were selected. The data were subjected to background correction and normalization using the R package “limma.” The pan-cancer TCGA dataset was downloaded from the UCSC (https://xenabrowser.net/) database.

### 2.4 Identification of DEGs

The 97 tumor samples were divided into G1, G2, and G3 groups based on clinical grading data. Gene expression differential analysis was performed using the limma package. DEGs were generated through pairwise comparisons among the three groups (i.e., G1 vs G2, G1 vs G3, G2 vs G3). DEGs were identified by the Wilcoxon rank sum test, with q = 0.05 and multiple change >1 after log 2 conversion as significant thresholds.

### 2.5 Gene ontology, Kyoto encyclopedia of genes and genomes and gene set enrichment analysis

To explore comprehensive information on large-scale gene data, GO and KEGG enrichment analyses were performed on DEGs using the clusterProfiler, enrichplot, and ggplot2 packages. Only terms with both p-values and q-values less than 0.05 were considered significantly enriched. The C2 (CP: KEGG_LEGACY, C5 (go.BP/CC/MF), and C7 (ImmuneSigDB) gene set v2023.2 collections were downloaded from the Molecular Signatures Database as target sets, and GSEA was performed using the GSEA-4.3.3 software downloaded from the Broad Institute. The whole transcriptome of 97 tumor samples was used for GSEA, and only gene sets with NOM p < 0.05 and FDR q < 0.06 were considered significant.

### 2.6 Survival analysis

Survival analysis was conducted using R language by loading the survival package. Ninety-seven tumor specimens with detailed survival time records were used for the survival analysis. The Kaplan-Meier method was employed to plot survival curves, and statistical significance was tested using the log-rank method, with p < 0.05 considered significant.

### 2.7 Univariate and multivariate Cox regression analyses

Univariate and multivariate Cox regression analyses were performed using the R language by loading the survival, forestplot, and rms packages to identify appropriate terms for constructing the nomogram and to plot the forest plot, showing the p-value, HR, and 95% CI for each variable.

### 2.8 TIC analysis

The R software package “CIBERSORT” was used to investigate the relationship of expression levels of single genes with the infiltration levels of immune-related cells. Screening conditions were: p-value < 0.05 was set and the results file was plotted against various immune cell correlations.

### 2.9 Relationship between single gene expression levels and immune checkpoint-related genes

Gene expression data was analyzed using the R software “limma” and “corrplot” together with a list of 47 immune checkpoint-related genes to assess whether there was a regulatory relationship between the immune checkpoint-related genes and the gene.

### 2.10 Statistical analysis

Mean ± SD were calculated and analyzed using SPSS software version 19.0 (IBM SPSS Statistics 19). Correlation between positive and negative and low and high PITX1 expression, and analyzed patient demographic and clinicopathologic variables with Fisher’s exact test. The Student–Newman–Keuls multiple range test was used to compare multiple groups. Individual pair-wise comparisons were made using the unpaired two-tailed Student’s t-test, and differences were deemed significant at p<0.05.

## 3 Results

### 3.1 Analysis procedure

**Figure 1:**
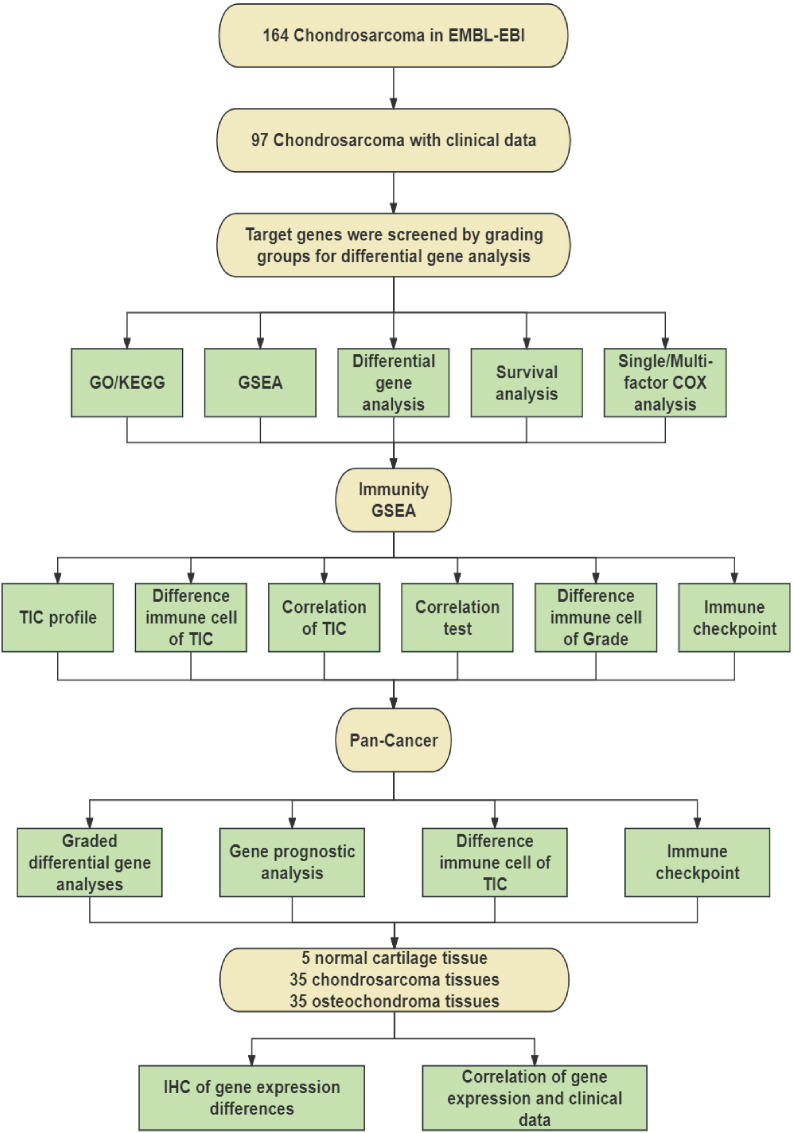
Schematic workflow of this study.

To identify differential genes associated with CHS grading, we screened 164 CHS samples downloaded from the EMBL-EBI database and selected 97 samples that included survival time and grading information. After processing, these samples were grouped based on their grading information. We conducted differential gene analysis to identify target genes, followed by GO and KEGG analyses of all DEGs. The common intersecting gene, PITX1, was subjected to GSEA, survival analysis, and univariate/multivariate Cox regression analysis to identify pathways influencing CHS grading. The process is shown schematically in **Figure 1**.

Subsequently, we employed immune GSEA analysis, immune cell infiltration assessment, and immune checkpoint analysis to investigate the impact of PITX1 on CHS immunity. Additionally, we conducted differential expression analysis, immune cell infiltration assessment, and immune checkpoint analysis on pan-cancer datasets to determine the effect of PITX1 on tumor-infiltrating cells (TICs) across various cancer types.

Finally, we utilized clinical samples that we collected to perform immunohistochemical staining to detect the expression of PITX1. By integrating this data with clinical information, our findings demonstrated that PITX1 can serve as a novel target for CHS grading, prognosis, and immunotherapy.

### 3.2 PITX1 expression correlates with CHS grading, division and proliferation

**Figure 2:**
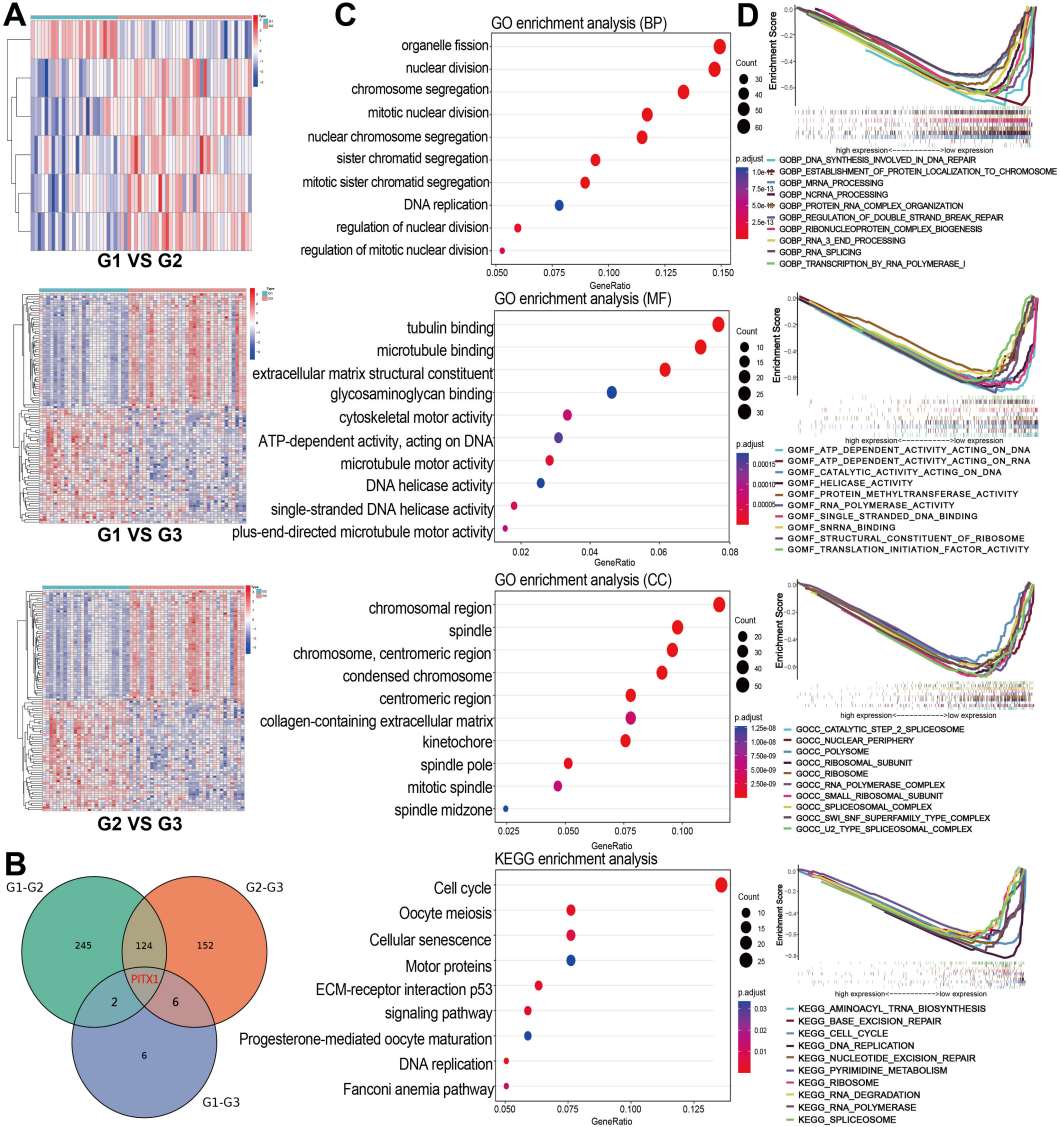
PITX1 expression correlates with CHS grade and proliferation **(A)** Heatmaps of DEGs were obtained by two-by-two comparisons between different grades of CHS. The line names in the heat map are the gene names, and the column names are the sample IDs not shown in the map. **(B)** Venn diagram showing the overlap of DEGs among the three groups, highlighting PITX1. **(C)** GO and KEGG enrichment analysis of all DEGs obtained from the three groups, the terms p and q < 0.05 were considered to be significantly enriched. **(D)** Gene sets enriched in samples with low PITX1 expression in the BP, CC, MF, and KEGG collections. Gene sets with unique color, and up-regulated genes located in the left approaching the origin of the coordinates, by contrast the down-regulated lay on the right of x-axis. Only several leading gene sets are displayed in the plot.

We obtained 97 samples by screening and processing 164 CHS samples downloaded from EMBL-EBI. The CHS samples were classified into Grade 1 (G1, n=25), Grade 2 (G2, n=39) and Grade 3 (G3, n=33) groups based on clinical grade. Genetic difference analyses comparing G1 vs. G2, G1 vs. G3, and G2 vs. G3 identified 6,376 total genes, among which were 279 DEGs (**Figure 2A**). By intersecting the DEG sets of all three groups, we identified a shared PITX1 gene that may be associated with tumor differentiation (**Figure 2B**).

Through GO and KEGG enrichment analysis, we identified that the DEGs were predominantly enriched in pathways related to cell division and proliferation. The Biological process (BP) analysis revealed that the DEGs were involved in processes, such as organelle fission, nuclear division and chromosome segregation. Cellular component (CC) enrichment showed DEGs were enriched in terms include “chromosomal region”, “mitotic spindle”, and “chromosome, centromeric region”. In terms of molecular function (MF), significant observations were made regarding activities, such as microtubule protein binding, cytoskeletal motor activity and extracellular matrix structural component activity. KEGG pathway analysis demonstrated DEGs were enriched in pathways involving cell cycle, oocyte meiosis and DNA replication (**Figure 2C**). Furthermore, GSEA analysis highlighted a significant enrichment of PITX1 in pivotal biological pathways, emphasizing its role in CHS progression (**Figure 2D**).

### 3.3 PITX1 is a protective factor and is expressed at low levels in high-grade CHS tumors

**Figure 3:**
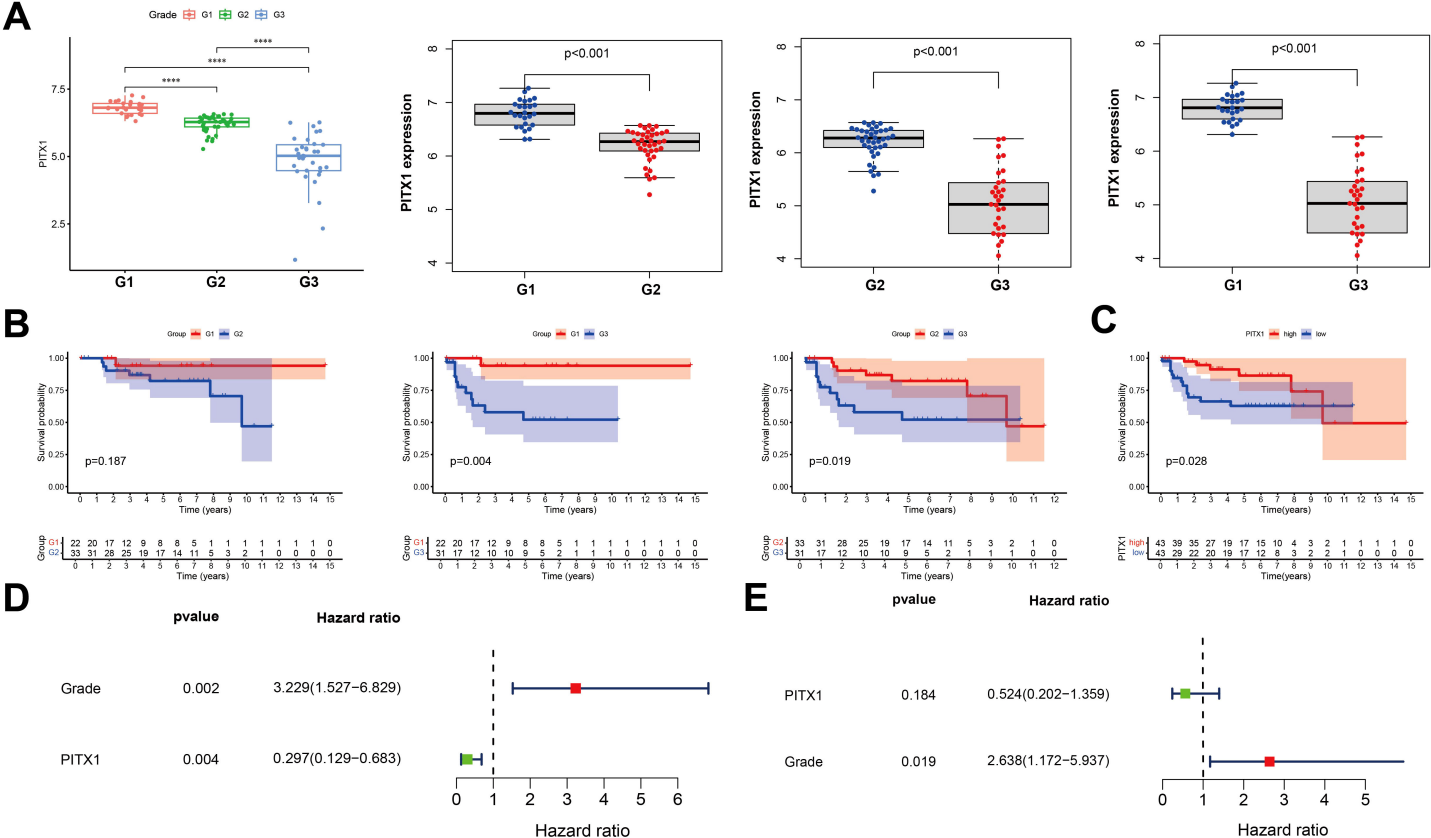
PITX1 is low-expression in high-grade and can be a protective factor in CHS **(A)** Box plot of PITX1 expression among groups of different tumor grades. **(B)** Expression of PITX1 was assessed using Kaplan-Meier survival analysis in patients with CHS. **(C)** Kaplan-Meier survival analysis. Patients were divided into high expressors or low expressors, depending on the comparison with the median expression level. P = 0.028 by log-rank test. **(D)** Forest plot showing univariate Cox regression analysis for grade and PITX1 expression with hazard ratios. **(E)** Forest plot showing multivariate Cox regression analysis with hazard ratios and p-values.

To investigate the correlation between PITX1 expression and CHS grade, survival and prognosis, we employed the Wilcoxon rank sum test. PITX1 expression was decreased in high-grade samples compared with low-grade samples (**Figure 3A**). OS analysis between different grades showed that although survival of G1 and G2 patients did not yield statistically significant differences (P > 0.05), overall, the low-grade group exhibited superior survival compared to the high-grade group (**Figure 3B**). Additionally, for survival analysis, 97 samples were categorized into PITX1 high-expression and PITX1 low-expression groups based on median PITX1 expression. The results demonstrated that the low-expression group had longer survival times compared to the high-expression group (**Figure 3C**). Both univariate and multivariate COX analyses demonstrated a hazard ratio (HR) < 1, indicating that low grade was associated with risk, with PITX1 showing a protective effect. However, the association between PITX1 and survival did not reach statistical significance in the multivariate analysis (**Figures 3D, E**). Overall, higher CHS grade was correlated with lower expression of PITX1. Furthermore, low expression of PITX1 was associated with poorer survival when considering its role as a protective factor.

### 3.4 PITX1 regulates infiltration of TICs and immune checkpoints in CHS

**Figure 4:**
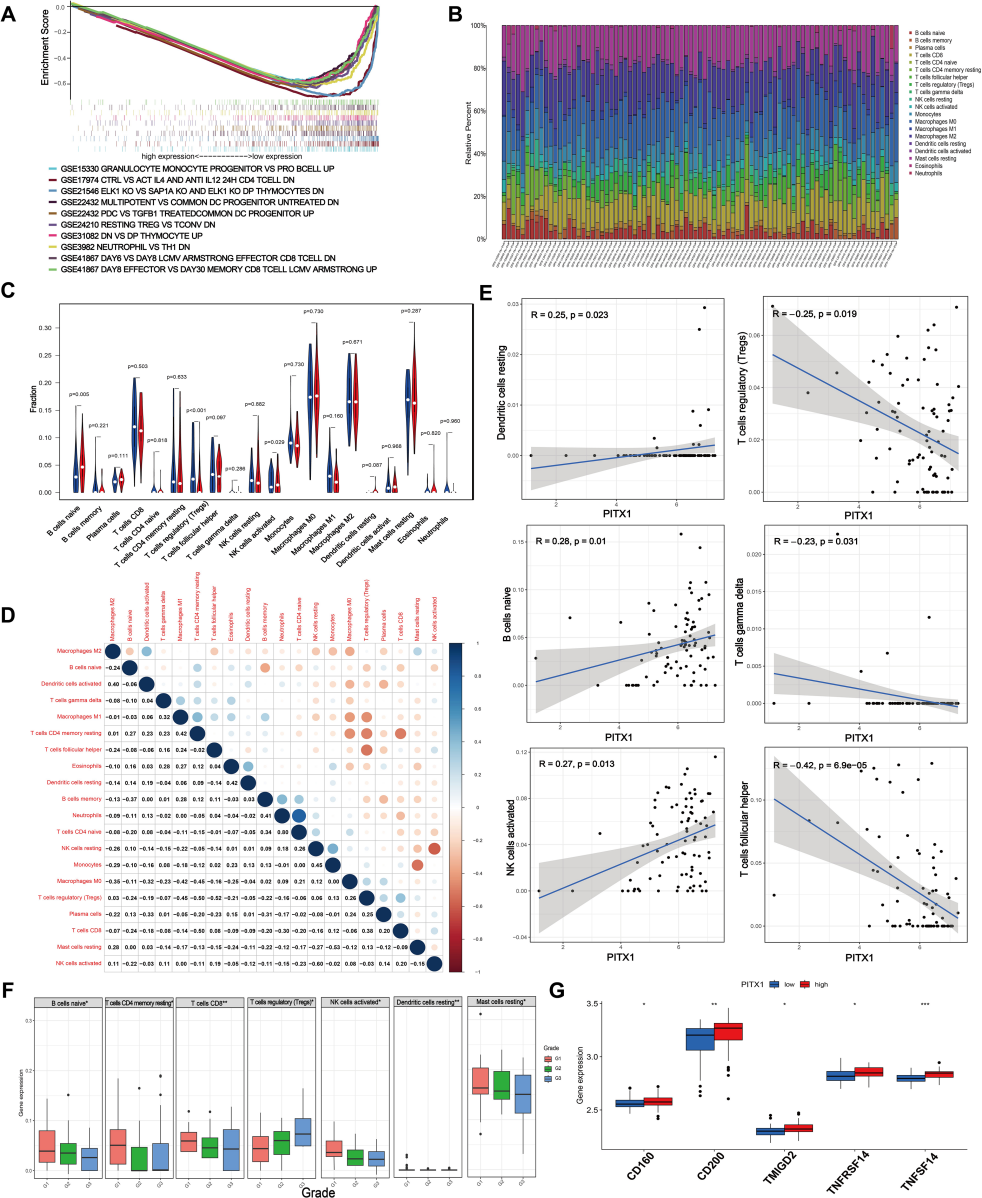
PITX1 regulates infiltration of TICs and immune checkpoints in CHS. **(A)** Gene sets enriched in low PITX1-expressing samples in immune collections. Gene sets with unique color, and up-regulated genes located in the left approaching the origin of the coordinates, by contrast the down-regulated lay on the right of x-axis. Only several leading gene sets are displayed in the plot. **(B)** Barplot showing the proportion of 20 types of TICs in CHS samples. Column names in the plot are the sample IDs. **(C)** Violin plot showing the ratio of differentiation of 20 kinds of immune cells between CHS samples with low or high PITX1 expression relative to the median of PITX1 expression level, and Wilcoxon rank sum was used for the significance test. **(D)** Heatmap showing the correlation between 20 kinds of TICs, and the number in each tiny box indicates the p-value of the correlation between two kinds of cells. The shade of each tiny color box represents the corresponding correlation value between two cells, and the Pearson coefficient was used for the significance test. **(E)** Scatter plot showing the correlation of 6 different TIC proportions and PITX1 expression (p < 0.05). The red line in each plot is a fitted linear model indicating the proportion of tropism of the immune cell along with PITX1 expression, and the Pearson coefficient was used for the correlation test. **(F)** Box showing the differential expression levels of different types of immune cells in different grades (p < 0.05). **(G)** Box showing the differential immune checkpoints based on PITX1 expression (p < 0.05).

To investigate the impact of PITX1 on the immune aspects of CHS, we conducted an immune-focused gene set enrichment analysis (GSEA) to examine the association between PITX1 expression and various types of immune cells and pathways (**Figure 4A**). A significant correlation existed between elevated levels of PITX1 and early progenitor cells, effector T cells, and specific dendritic cell populations. Conversely, decreased levels of PITX1 were observed in differentiated or resting immune cell states. These results suggest that PITX1 may play a crucial role in modulating immune responses, thereby influencing the development and function of diverse immune cells.

To further validate the correlation between PITX1 expression and the immune microenvironment, we utilized the CIBERSORT algorithm to analyze the proportions of tumor-infiltrating immune subgroups and generated a profile encompassing 20 different types of immune cells in CHS samples (**Figure 4B**). Differential and correlation analyses indicated that three types of tumor-infiltrating cells exhibited differential expression patterns that were significantly associated with PITX1 expression levels (naive B cells, follicular helper T cells, activated NK cells) (**Figures 4C–E**). Notably, naive B cells and activated NK cells had positive correlations with PITX1 expression, while follicular helper T cells had a negative correlation. These findings provide additional support for the hypothesis that PITX1 levels influence immunological activity within the tumor microenvironment (TME).

Subsequently, we conducted a differential, by tumor grade, of immune subgroups infiltrating CHS. Elevations in the infiltration levels of naive B cells, resting memory CD4^+^ T cells, CD8^+^ T cells, activated NK cells, resting dendritic cells, and resting mast cells occurred with increasing grade. Conversely, regulatory T cell (Treg) infiltration decreased as tumor grade increased (**Figure 4F**). Furthermore, immune checkpoint analysis revealed upregulation of CD160, CD200, TMIGD2, TNFRSF14, and TNFSF14 in the PITX1 high-expression group (**Figure 4G**). In summary, these results suggest that PITX1 can influence the infiltration of TICs and modulate immune checkpoints in CHS.

### 3.5 PITX1 can act as a tumor grade marker and diagnostic marker for immunotherapy in multiple cancers

**Figure 5:**
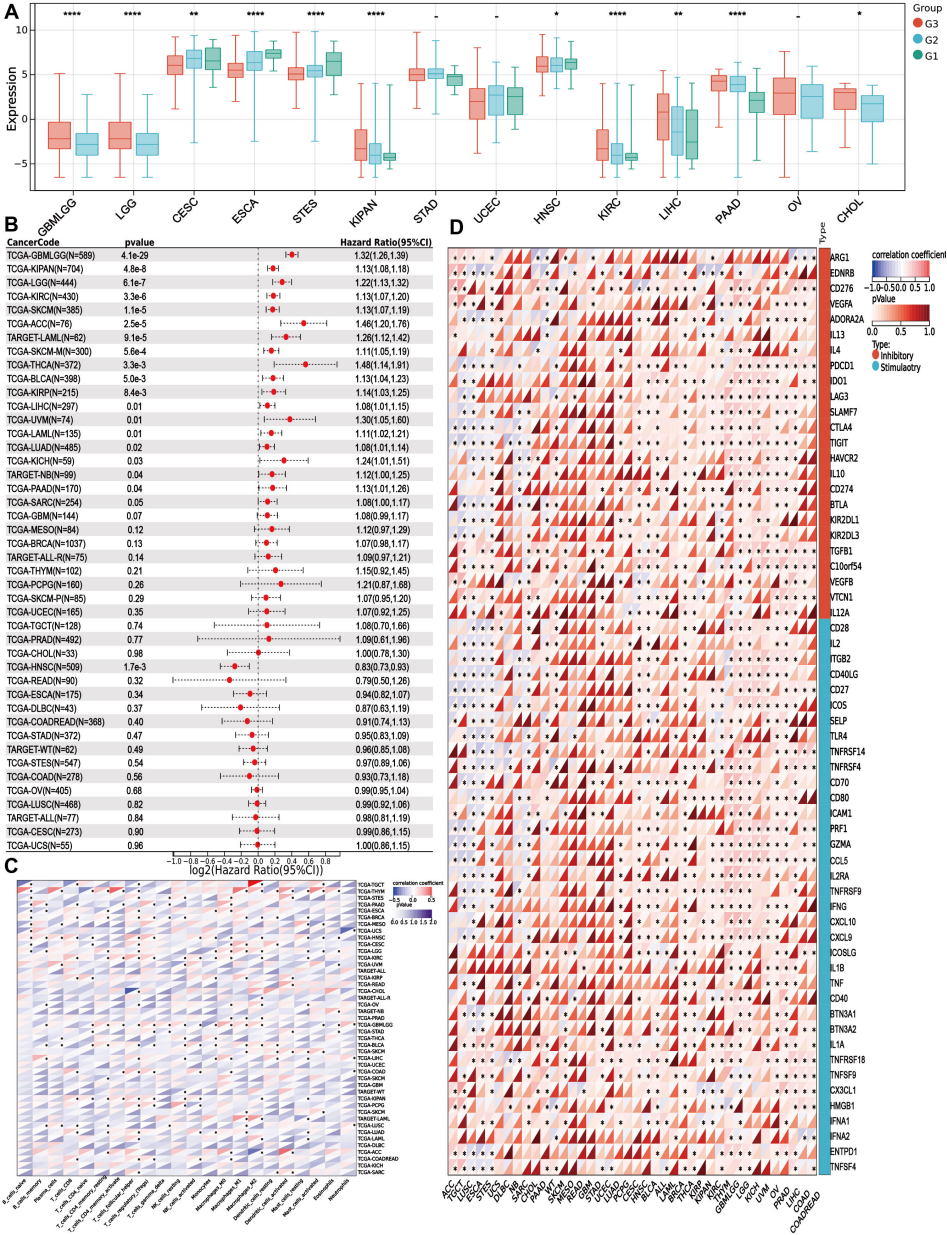
PITX1 can act as a marker for tumor grade and as an immunotherapy target in multiple cancers **(A)** Box plots showing the difference in PITX1 expression among different grades in 14 cancers. And Wilcoxon rank sum were used for the significance tests. **(B)** Forest plots showing the results of univariate COX regression analysis of PITX1 expression in 39 cancer tissues. **(C)** Heatmap showing the correlation between 22 TICs and 39 cancer tissues, with the number in each small box representing the p-value of the correlation between the two. The depth of each small color box represents the corresponding correlation value between the two cells. The Pearson coefficient was used to determine significance. **(D)** Heatmap showing the correlation between PITX1 expression and the expression of multiple immune checkpoint genes across different cancer types. (* p<0.05, **p<0.01, *** p<0.001).

To explore whether PITX1 exhibits a similar tumor grade-related characteristic in other cancer types as observed in CHS, we analyzed tumor grade and gene expression across various cancer types. We identified significant variations in PITX1 expression across 14 tumor types (**Figure 5A**). However, only CESC, ESCA, STES, UCEC, and HNSC exhibited concordant tumor grade-related expression patterns similar to CHS. Notably, low PITX1 expression was associated with poorer prognosis exclusively in HNSC (**Figure 5B**), whereas 19 other cancer types demonstrated improved prognosis with low PITX1 expression. Our pan-cancer analysis revealed significant associations between PITX1 expression and immune infiltration across 39 cancer types (**Figure 5C**), indicating its role in regulating TIC. Furthermore, immune checkpoint analysis revealed a correlation between PITX1 expression and expression of pivotal immune checkpoint genes across various cancer types (**Figure 5D**), indicating that PITX1 could serve as a promising diagnostic marker for immunotherapy.

### 3.6 PITX1 is associated with differentiation and metastasis in CHS patients

**Figure 6:**
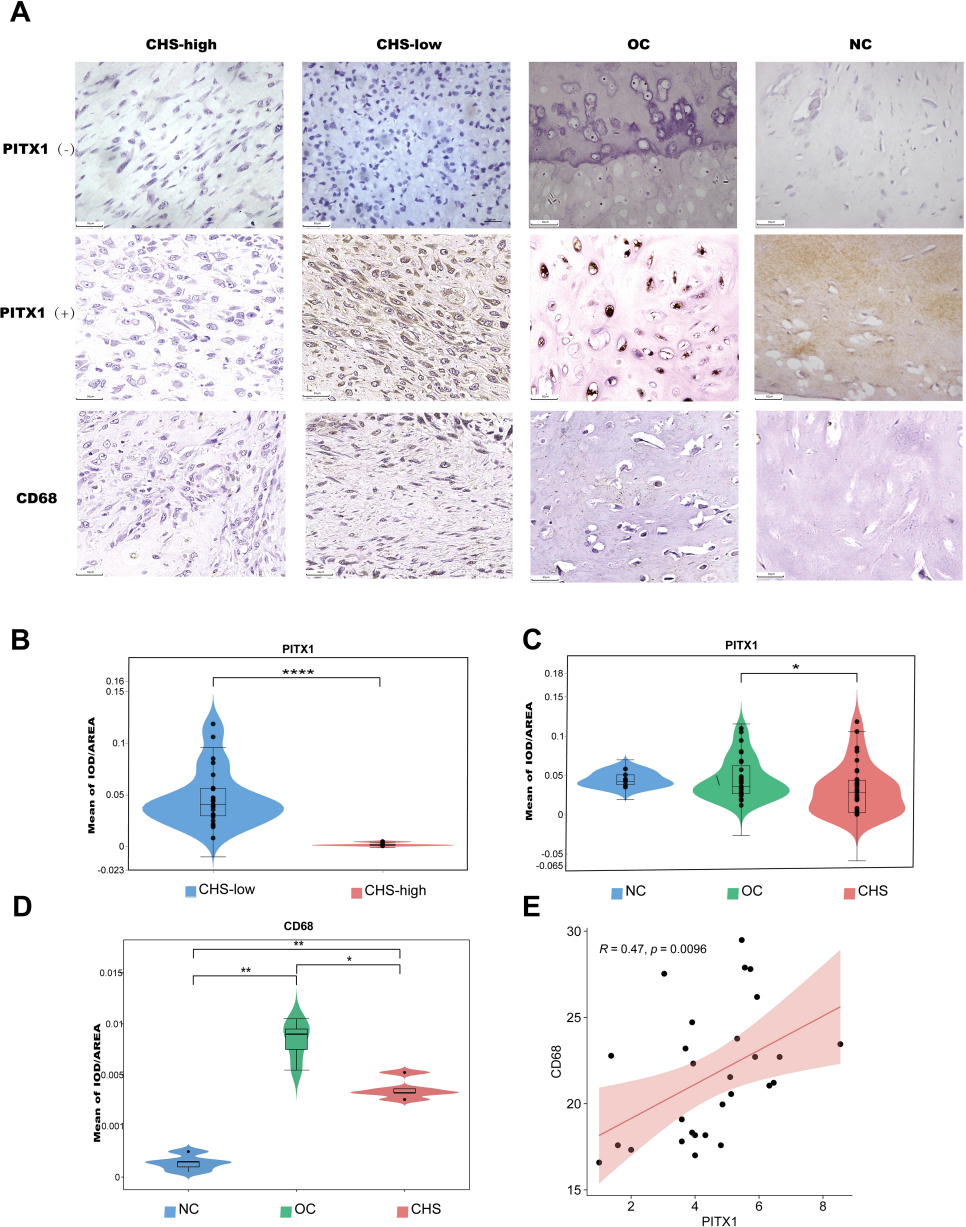
PITX1 is associated with differentiation and metastasis in CHS patients. **(A)** The expression of PITX1 in patient tissues was detected by IHC. First column: high-grade CHS weakly expressing/not expressing PITX1,CD68; second column: low-grade CHS strongly expressing/not expressing PITX1,CD68; third column: OC strongly expressing/not expressing PITX1,CD68; fourth column: normal cartilage tissue strongly expressing/not expressing PITX1,CD68. **(B)** Mean intensity of PITX1 expression in CHS (low-grade vs high-grade) (****p<0.001). **(C)** Comparison of the mean of IOD in CHS, OC and normal cartilage (* p<0.05). **(D)** Comparison of the mean of IOD in CHS, OC and normal cartilage (* p<0.05, **p<0.001). **(E)** Scatterplot showing the correlation between CD68 and PITX1 expression (p < 0.05). The red line in each plot is a fitted linear model indicating the relationship between CD68 and PITX1 expression, and the correlation test used the Pearson coefficient.

Immunohistochemistry identified the expression of PITX1 and CD68 (macrophages) in CHS, OC, and normal cartilage tissues.PITX1 and CD68 were expressed in all cartilage tissues as well as in OC and CHS tissues. However, strong nuclear and cytoplasmic staining for PITX1 was observed in normal cartilage tissues and OC cases, whereas weak cytoplasmic staining was observed in CHS cases.CD68 was expressed at a low level in cartilage tissues, whereas it was strongly expressed in OC cases, and at a lower level in the highly differentiated group of CHS compared to the less differentiated group (**Figure 6A**). Based on integral optical density (IOD), the cases were divided into PITX1-strong and PITX1-weak subgroups. Strong expression of PITX1 was observed to be significantly strong in all normal tissue specimens, with a statistically significant increase in 85.7% (30/35) of OC tissue specimens (p<0.05) and a moderate increase in 54.3% (19/35) of CHS tissue specimens (p<0.05). In addition, the mean IOD of PITX1 in low-grade CHS was 0.0470 ± 0.0280, which was significantly higher than that of high-grade CHS tissues (0.0019 ± 0.0016) (**Figure 6B**). However, there was no difference among normal cartilage (0.0449 ± 0.0095), OC (0.0451 ± 0.0256) and the average of high-grade and low-grade CHS tissues (0.0315 ± 0.0313) (**Figure 6C**). Macrophage (CD68) infiltration differed between normal cartilage (0.00039 ± 0.0005), OC (0.0085 ± 0.0016), and the mean of high-grade versus low-grade CHS tissue (0.0042 ± 0.0012) (**Figure 6D**). Meanwhile, in CHS, the expression of PITX1 was positively correlated with macrophage infiltration (R=0.47, P=0.0096), which was the same as the result of previous bioinformatics analysis (**Figure 6E**).

We then investigated the correlation between PITX1 expression and clinicopathologic parameters in OC and CHS patients, including age, gender, tumor size, and tumor location. No significant correlation between PITX1 expression and age, gender, or tumor location was observed. However, a statistically significant association was observed between PITX1 expression and both tumor differentiation (p<0.001), as well as metastasis (p=0.042) (**Table 2**). On the other hand, no significant correlation was found between clinical parameters and PITX1 expression in OC patients (**Table 3**). These findings suggest that downregulation of PITX1 expression is particularly associated with high-grade CHS and correlates with metastasis in patients, as well as with macrophage infiltration.

**Table 2:** Correlation between clinical parameters and PITX1 expression in CHS.

Clinical parameters	PITX1 expression in chondrosarcoma (N, %)		
	WEAK 16 (45.7)	STRONG 19 (54.3)	P
Age			0.865
<30 y (7, 20%)	3	4	
≥30 y (28, 80%)	13	15	
Gender			0.508
Male (22, 62.9%)	11	11	
Female (13, 37.1%)	5	8	
Differentiation			0.000
High (12, 34.3%)	12	0	
Low (23, 65.7%)	4	19	
Location			0.103
Pelvis (7, 20%)	6	1	
Lower limb (12, 34.3%)	3	9	
Upper limb (4, 11.4%)	1	3	
Skull (7, 20%)	3	4	
Others (5, 14.3%)	3	2	
Metastasis/Recurrence			0.042
Yes (6, 17.1%)	5	1	
No (29, 82.9%)	11	18	

**Table 3:** Correlation between clinical parameters and PITX1 expression in OC.

Clinical parameters	PITX1 expression in osteochondroma (N, %)		
	WEAK 5 (14.3)	STRONG 30 (85.7)	P
Age			0.155
<30 y (26, 74.3%)	5	21	
≥30 y (9, 25.7%)	0	9	
Gender			0.167
Male (18, 51.4%)	4	14	
Female (17, 48.6%)	1	16	
Tumor size			0.782
<2.5 cm (16, 45.7%)	2	14	
≥2.5 cm (19, 52.3%)	3	16	
Location			0.772
Lower limb (24, 68.6%)	4	20	
Upper limb (9, 25.7%)	1	8	
Others (2, 5.7%)	0	2	
Metastasis			–
Yes 0	0	0	
No (35, 100%)	5	30	

## Discussion

Chondrosarcoma has a low incidence, is a relatively rare tumor, and has always been less studied than other tumors. This has resulted in relatively few biomarkers for chondrosarcoma (**Table 4**). We show that decreased PITX1 is associated with high-grade CHS and elevated expression correlates with good prognosis, serving as a protective factor for CHS. Although, the prognostic results of PITX1 did not reach statistical significance in multivariate Cox analysis, univariate COX analysis proved that low expression was associated with poorer prognosis, which may be due to insufficient sample size or other multifactorial interventions, but its overall results can prove that low expression of PITX1 is associated with poorer prognosis, which is clinically significant. Notably, immune analysis demonstrates a positive correlation between PITX1 and TIC in the context of CHS, as well as pan-cancer analysis. Finally, we found that PITX1 expression levels are correlated with CHS grade, tumor differentiation and metastasis status in our patient cohort. These results suggest that PITX1 may serve as a valuable biomarker for prognosis and therapeutic targeting in CHS patients.

**Table 4:** Biomarkers in OC.

Gene	Expression	Ref
DDX10	High	(53)
BYSL	High	(53)
WDR12	High	(53)
MMP7	High	(54)
IDH1	High	(55)
KDELR1	High	(56)
ADAM8	High	(57)
INSM1	High	(58)
LRF	High	(59)
COL1A2	High	(60)
CCL5	High	(61)
CDK8	High	(62)
EEF1A1	High	(62)
NTN1	High	(62)
NAMPT	High	(63)
CD44V6	High	(64)
EZH2	High	(65)
SIRT1	High	(66)
IDH2	High	(67)
CHRNA6	High	(68)
LINC00665	High	(69)
SF2523	Low	(70)
PLCD1	Low	(71)
TXNIP	Low	(72)
PRKCZ	Low	(73)
Ugonin	Low	(74)

PITX1 plays an important role in the normal development of the limbs and nervous system in vertebrate embryos (16, 23, 24). PITX1 has been shown to be associated with a variety of cancers (**Table 5**). For example, PITX1 has been shown to be down-regulated and to provide a positive prognosis in cancers such as osteosarcoma, oral squamous cell carcinoma, gastric cancer, malignant melanoma, esophageal, colorectal cancer, and lung cancer. However, its high expression correlates with negative prognosis in breast, prostate and squamous cell carcinoma of the skin (25). PITX1 has a regulatory effect on various functions in cancers. For example, our laboratory previously found that PITX1 expression is down-regulated in osteosarcoma and correlates with patient survival and lung metastasis, and that PITX1 suppresses OS cell proliferation and metastasis by downregulating LINC00662. Moreover, LINC00662 can be packaged into OS cell-derived exosomes to mediate M2 macrophage polarization to promote OS metastasis via CCL22 (22, 26). In breast cancer cells, PITX1 can directly bind to the p53 promoter and activate p53, which in turn activates stress-related signaling pathways, involving DNA damage, oncogene activation, hypoxia and nutritional deficiency, leading to cell cycle arrest or apoptosis (27, 28). In colon cancer, PITX1 inhibits tumorigenesis and proliferation by downregulating the RAS pathway and upregulating the p53 pathway (17, 27). In gastric cancer, PITX1 induces apoptosis and inhibits cell proliferation by binding to the apoptosis-related gene PDCD5 promoter and blocking the cell cycle in G1/S phase (29). Similarly, in melanoma PITX1 can bind to RE1 (-592/-588) and RE3 (-520/-504) within the SOX9 promoter region, promoting SOX9 expression, apoptosis and inhibiting melanoma cell proliferation (30). However, studies on PITX1 as a prognostic marker for cancer grade are still relatively lacking. Currently, associations with grade have only been observed in lung, prostate, gastric and head and neck squamous cell carcinomas (20, 31–33). Our findings discovered for the first time that PITX1 correlates with grade in chondrosarcoma, with lower expression at higher grades. In addition, our findings also discovered that PITX1 is associated with the immune pathway.

**Table 5:** PITX1 expression in cancer.

Cancer type	PITX1 expression	Ref
Bladder Cancer	Low	(1, 17)
Prostate Cancer	Low	(17, 32)
Colon Cancer	Low	(17, 75)
Esophageal Cancer	Low	(19, 76)
Breast Cancer	High	(21, 28)
Gastric Cancer	Low	(29)
Leukemia	High	(77)
Liver Cancer	Low	(78)
Melanoma	Low	(79)
Osteosarcoma	High	(22)
Skin	High	(80)
Head and Neck Cancer	High/Low	(81, 82)
Kidney Cancer	High/Low	(83, 84)
Lung Cancer	High/Low	(20, 85–87)

Despite numerous studies of the tumor immune microenvironment, the effects of PITX1 on TICs and immune checkpoints in tumors have not been reported, and studies on the immune status of CHS remain limited (34). Currently, researchers have provided a comprehensive integrated immune profile of conventional CHS, based on CyTOF, multicolor flow cytometry, WES, radiological, and pathological assessments. This has made a significant contribution to predicting the immune status of CHS and guiding immunotherapy (35). Researchers have found that immune infiltrating cells (particularly PD-1-positive T cells) and HLA Class I expression are present in PD-L1-positive dedifferentiated CHS (36). Additionally, considering the importance of TIL localization in the response to immunotherapy for solid tumors, researchers have found that in some low-grade tumors, immune cells are mainly located at the periphery of cartilage islands. In some structurally disorganized and highly aggressive dedifferentiated CHS, immune cells are found in close proximity and intermixed with tumor cells. This may be due to the dense hyaline ECM hindering tumor infiltration (37, 38). In chondrosarcoma, our findings discovered that only the infiltration of naive B cells, activated NK cells, and regulatory T cells correlated and differed with PITX1 expression, with naive B cells and activated NK cells being positively correlated, and regulatory T cells being positively correlated with PITX1 expression. The higher the CHS classification, the lower the number of CD8^+^ T cells, memory resting CD4^+^ T cells, follicular helper T cells, activated NK cells, and resting dendritic cells, and the higher the number of follicular helper T cells and resting mast cells. It is well known that activated NK cells and naïve B cells are important immune cells for tumor killing. Naïve B cells can recognize tumor-associated antigens through their B cell receptor (BCR), initiate an immune response, differentiate into plasma cells, secrete antibodies, and label tumor cells. Activated NK cells can recognize tumor cells labelled by antibodies through the Fc receptor (CD16) on their surface, and either kill them directly through the release of perforins and granzymes, or kill the tumor cells through the release of cytotoxic molecules. In addition to directly killing tumor cells, activated NK cells can also secrete a variety of cytokines, such as IFN-γ, which can directly inhibit the growth of tumor cells and enhance the anti-tumor activity of other immune cells (e.g., macrophages and T cells) (39–44). Tregs play a major role in tumors in promoting an immunosuppressive environment that allows tumor cells to survive and proliferate, and it also reduces antigen presentation and effector T-cell activity by inhibiting dendritic cell function. It has also been shown that Tregs promote tumor angiogenesis through the secretion of pro-angiogenic factors, such as VEGF, which provide the nutrients and oxygen needed for tumor growth (45–47). These three TICs have yet to be associated with PITX1 in tumors. Our results indicate that PITX1 may potentially inhibit tumor progress and kill tumors by recruiting NK and B cells while reducing Treg infiltrate in CHS.

Our results indicate that immune checkpoints CD160, CD200, TMIGD2, TNFRSF14, TNFSF14 are up-regulated in the PITX1 high-expression group. It has been reported that CD200, TMIGD2 and TNFSF14 can facilitate anti-tumor immune responses by enhancing T and NK cell activity in multiple myeloma and hematologic malignancies. In addition TNFSF14 can also influence tumor growth by modulating tumor angiogenesis and microenvironment in gliomas. However, CD160 and TNFRSF14 are highly expressed in a variety of tumors (e.g. chronic lymphocytic leukemia and multiple myeloma) and promote tumor evasion of immune surveillance by regulating NK and T cell activity (48–52). There have been no findings of PITX1 associated with CD160, CD200, TMIGD2, TNFRSF14, and TNFSF14 immune checkpoints, so our results identified for the first time an association between PITX1 and immune checkpoints. In summary, PITX1 may influence the development of CHS by modulating expression of the aforementioned immune checkpoints, which in turn affects the activity of T cells, B cells, and NK cells, leading to altered TICs and the development of immune evasion in chondrosarcoma.

Progress in CHS treatment has been slow. Therefore, researchers must continue to explore new therapeutic strategies at the molecular and clinical levels. Despite our finding that PITX1 can serve as a new target for CHS grade, metastasis, and immunotherapy, this study has certain limitations. For instance, our bioinformatics analysis and immunohistochemistry were based solely on data from public databases and a limited number of samples. We did not employ additional methods to validate our findings. Moreover, the lack of patient survival data hindered us from establishing a correlation between PITX1 expression and patient outcomes. The regulatory mechanisms underlying PITX1 expression in human CHS remain unknown. Furthermore, future studies involving larger patient populations are needed to thoroughly evaluate the predictive significance of PITX1 as a novel biomarker for CHS diagnosis and prognosis. In conclusion, this study identifies PITX1 as a novel molecular marker, particularly applicable for improving the diagnosis and prognosis of high-grade CHS.

## Data Availability

The raw data supporting the conclusions of this article will be made available by the authors, without undue reservation.
